# Thermal Conductivity of Ordered Porous Structures Coupling Gas and Solid Phases: A Molecular Dynamics Study

**DOI:** 10.3390/ma14092221

**Published:** 2021-04-26

**Authors:** Dong Niu, Hongtao Gao

**Affiliations:** Institute of Refrigeration & Cryogenics Engineering, Dalian Maritime University, Dalian 116026, China; niudong@dlmu.edu.cn

**Keywords:** thermal conductivity, porous structures, molecular dynamics simulation, Green−Kubo method

## Abstract

Heat transfer in a porous solid−gas mixture system is an important process for many industrial applications. Optimization design of heat insulation material is very important in many fields such as pipe insulation, thermal protection of spacecraft, and building insulation. Understanding the micro-mechanism of the solid−gas coupling effect is necessary for the design of insulation material. The prediction of thermal conductivity is difficult for some kinds of porous materials due to the coupling impact of solid and gas. In this study, the Grand Canonical Monte Carlo method (GCMC) and molecular dynamics simulation (MD) are used to investigate the thermal conductivity for the ordered porous structures of intersecting square rods. The effect of gas concentration (pressure) and solid−gas interaction on thermal conductivity is revealed. The simulation results show that for different framework structures the pressure effect on thermal conductivity presents an inconsistent mode which is different from previous studies. Under the same pressure, the thermal conductivity is barely changed for different interactions between gas and solid phases. This study provides the feasibility for the direct calculation of thermal conductivity for porous structures coupling gas and solid phases using molecular dynamics simulation. The heat transfer in porous structures containing gas could be understood on a fundamental level.

## 1. Introduction

Porous materials are widely used in many areas, such as building insulation, gas storage and separations, aerospace, and so on. Heat transfer in porous materials containing the gas phase is an important process that cannot be completely understood on the fundamental level, especially for nanoporous materials. Accurate prediction of thermal conductivity for nanoporous materials is extremely necessary for the application.

A great number of theoretical models for the thermal conductivity were developed in previous studies [1,2,3,4,5,6] by considering the effect of gas, solid, and radiation in nonporous material in which some deviations exist compared with the experimental value to some extent. Due to the extremely complicated structures and solid−gas coupling effect, many empirical parameters are needed in the theoretical model which is hard to determine. Numerical simulation is a possible choice to directly obtain the properties of nanoporous materials. The lattice Boltzmann method was used to study the phonon heat transfer in the spherical segment of nano silica aerogel grains [7]. The simulation results indicated that the temperature distribution in the silica aerogel grain depends strongly on the size, and the phonon scattering at the boundary surfaces becomes more prominent when grain size decreases. Li et al. [8] developed a modified model for predicting the gaseous thermal conductivity in nanoporous materials based on the Direct Simulation Monte Carlo (DSMC) method. The results showed that the modified model has a higher accuracy without complex calculations and assumptions. The size effects on gas thermal conductivity were studied with the DSMC method [9]. There exists an obvious temperature jump at the boundary and the thermal conductivity tends to decrease when the Knudsen number increases from 0.01 to 0.1. Zhao et al. [10] obtained the gaseous thermal conductivity of nitrogen using the three-dimensional DSMC method with the variable soft sphere collision model. The results are consistent with the experimental data but much higher than those by the Eucken relation, especially at high temperature. The nanoporous aerogels were reconstructed by an improved three-dimensional diffusion-limited cluste-cluster aggregation (DLCA) method in which the contribution of the solid−gas coupling heat transfer to the gas−contributed thermal conductivity was quantified [11]. A modified lattice Boltzmann method [12] was developed to predict the effective thermal conductivity of nanoporous aerogel materials which introduced an additional coefficient to regulate the difference in thermal conductivity between solid and gas phases and guaranteed a converged solution. Fang et al. [13] used the lattice Boltzmann method to solve the conduction−radiation equation for predicting the effective thermal conductivity. The simulation results showed that the effective thermal conductivity of the pure nanoporous aerogel increases rapidly with temperature and is greatly suppressed if additives are doped. The thermal conductivity of nanoporous film and nanocomposite was numerically studied by solving the phonon Boltzmann transport equation with the frequency-dependent model [14]. The local angle between heat fluxes and the local heat flux was introduced and all the results showed that the nanostructured material with a larger average angle would have a lower thermal conductivity. Tang et al. [15] investigated the thermal conduction of nanoporous silicon thin film using the Discrete Ordinates Method (DOM). The effects of the thickness, porosity and porous structure were considered. The numerical results showed that the nanopores are able to reduce the thermal conductivity of the silicon thin film sharply due to the phonon boundary scattering.

Molecular dynamics simulation is also an effective method to solve the nanoscale gaseous and solid heat transfer problems. Coquil et al. [16] employed the non-equilibrium molecular dynamics simulation to predict the thermal conductivity of amorphous nanoporous silica for the first time. The simulation results revealed that the thermal conductivity of nanoporous silica at room temperature is independent of pore size and depends only on porosity. Ng et al. [17] investigated the thermal conductivity of nanoporous aerogel samples at various densities through negative pressure rupturing of dense silica samples. The results indicated that a power-law fit of thermal conductivity varies almost linearly with density, in which the decreasing density and increasing porosity would lead to a linear decrease for thermal conductivity. The heat transport in secondary particles chain of nanoporous silica aerogel was studied using molecular dynamics simulation [18]. The heat transport was suppressed when the contact length or defect concentration increased, and the constrain effect was much more obvious when the contact length fraction was in the small range. The heat transfer characteristics of nanoscale-confined gas were investigated by the molecular dynamic simulation [19]. The effect of wall force field, wall stiffness, and the wall−gas interaction potential strength on the effective thermal conductivity were considered. Babaei and Wilmer [20] investigated the mechanisms of heat transfer in the porous crystal−gas mixture system using the molecular dynamics simulation. The study revealed that the thermal conductivity of the system is dominated by the crystal, which is reduced as the concentration of gas in the pores increases. The decreased conductivity associated with increased gas concentration is due to the phonon scattering of the crystal caused by the interactions with gas molecules.

Although a large number of works have been carried out on the thermal conductivity of nanoporous material, the theoretical model is particularly difficult to be used to directly describe the detailed gas−solid coupling interaction, and most simulation works focus only on a single component, the solid or the gases. In this work, we perform the equilibrium molecular dynamics simulation to study the thermal conductivity of ordered porous structures coupling gas and solid phases. The effect of pressure and gas−solid interaction on the thermal conductivity is considered.

## 2. Materials and Methods

**Figure 1:**
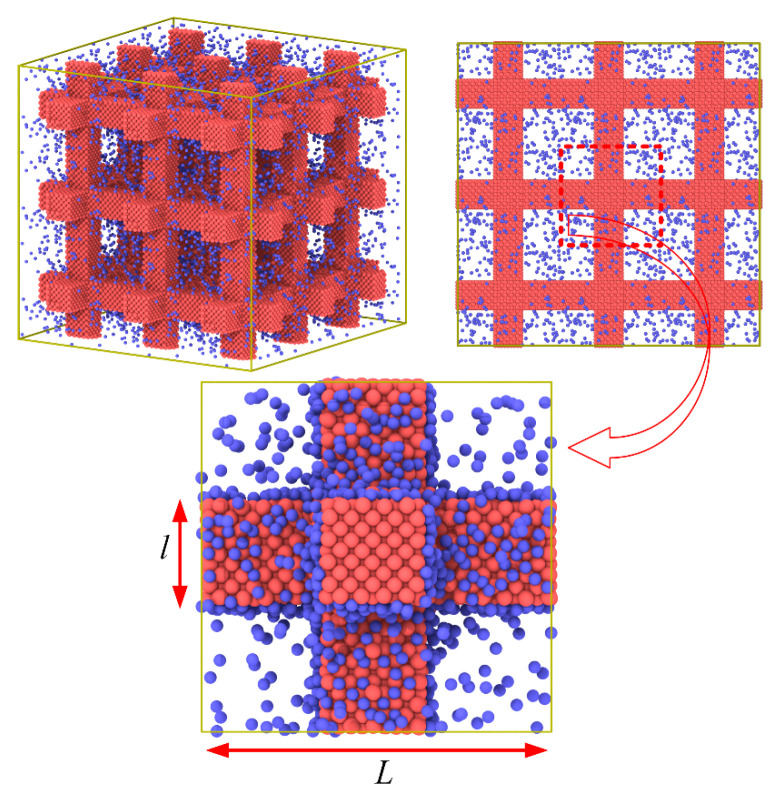
The setup of the simulation system.

Molecular dynamics simulation in this work is performed adopting the LAMMPS package [21]. The simulation cell including the gas and solid is obtained from the ordered porous structures of intersecting square rods as shown in Figure 1. The periodic boundary condition is used in all directions. The argon is chosen as the solid phase of the ordered porous structures filled with the gas of helium at 20 K. Solid argon is a face-centered cubic crystal with a lattice constant *S* = 5.4 Å. The Lennard−Jones 12–6 potential, *E* = 4*ε*[(*σ*/*r*)^12^ − (*σ*/*r*)^6^], is used to describe the interaction between atoms and truncated at a cutoff radius of 14 Å. The detailed parameters used in our simulation are listed as in Table 1. The NVT ensemble which keeps the number of atoms, volume, and temperature constant is used with a Nosé−Hoover thermostat at 20 K with a time step of 5 fs.

The thermal conductivity of the nanoporous system is calculated using the Green−Kubo formula

(1) $$\lambda =\frac{V}{k_{b}T^{2}}\int_{0}^{\infty }\langle j(0)j(t)\rangle dt$$

where *k_b_* is the Boltzmann constant, *V* is the volume of the simulation cell and the angular brackets denote the average over time. The microscopic heat flux *j* is obtained from the following equation

(2) $$j(t)=\frac{1}{V}\left(\sum_{i}v_{i}\varepsilon_{i}+\frac{1}{2}\sum_{i}\sum_{j,j\neq i}r_{ij}\left(v_{i}\cdot F_{ij}\right)\right)$$

where *v_i_* is the velocity of atom *i* and *F_ij_* is the force on atom *i* from the atom *j*. The *ε_i_* in the first term of Equation (2) is the per-atom energy including the potential and kinetic energy.

To investigate the thermal conductivity of the nanoporous system under different pressures, all simulations are performed in two stages. In the first stage, the Grand Canonical Monte Carlo simulation is used to determine the gas content for different pressures. The algorithm produces a grand-canonical ensemble where the gas atoms can be displaced, deleted, or created in the simulation box. During this stage, a great number of attempts for gas atoms to be inserted and deleted are carried out in every step. The insertion or deletion for gas atoms is equal probability anywhere judged by the usual criteria of the Grand Canonical Monte Carlo algorithm. The gas of helium is treated as an ideal gas and the chemical potential *μ* can be defined using the pressure *P* given by *μ* = *k_b_T*ln(*Pφ*/*k_b_T*), where *φ* is the fugacity coefficient. The number of gas atoms is averaged for the last 400,000 steps as the gas content for different pressures. In the second stage, the simulation cell including the solid and gas firstly achieves the equilibrium state in the NVT ensemble at 20 K. Then, the thermal conductivity of the nanoporous system is calculated using the Green−Kubo formula. The heat flux vector is recorded every 5 timesteps in the NVE ensemble for 5 × 10^7^ timesteps. The correlation time is 10,000 timesteps. For all cases, we performed 5 independent simulations with different random seeds for the velocity distribution of atoms. The averaged value of the 5 simulations is used for the thermal conductivity predictions.

**Figure 2:**
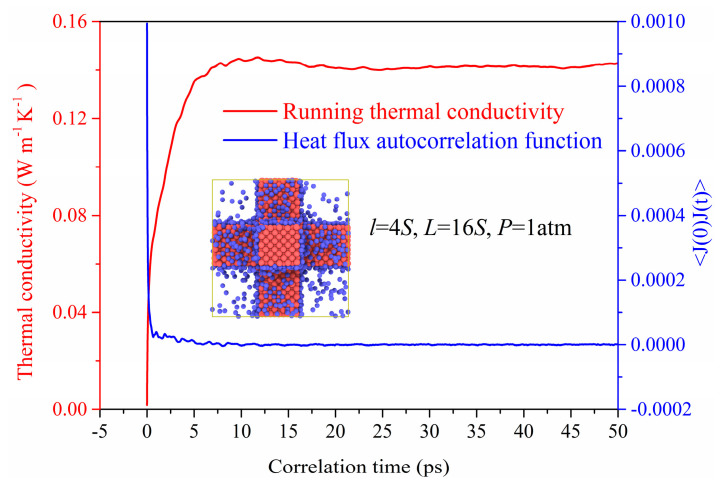
The HFACF and running thermal conductivity.

Figure 2 shows the heat flux autocorrelation functions (HFACF) responding to Equation (1) for the nanoporous system. We can find that the HFACF gradually approaches zero at about 10 ps, which also proves that the correlation time of 50 ps used in our simulation is long enough to obtain the stable thermal conductivity. The running thermal conductivity is obtained based on the HFACF which would stabilize around 0.14 W m^−1^ K^−1^ after 20 ps.

## 3. Results

### 3.1. The Pressure Effect

**Figure 3:**
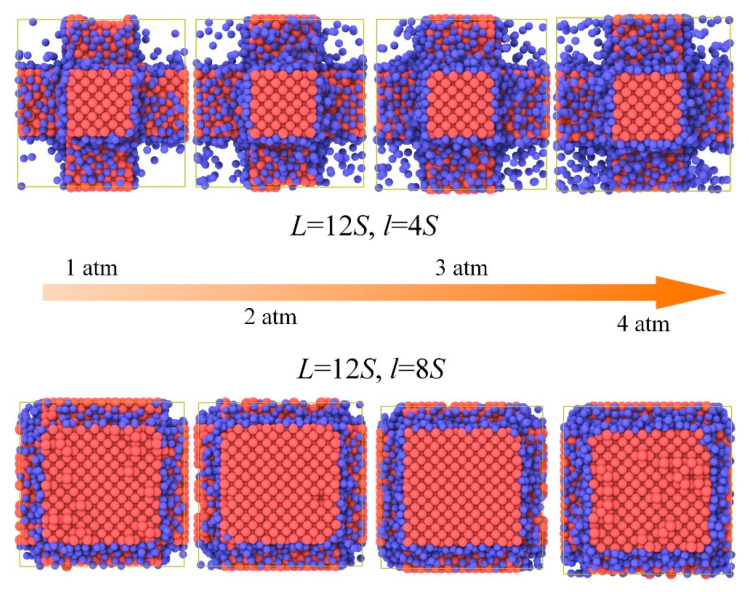
Gas atoms distribution in the nanoporous systems.

**Figure 4:**
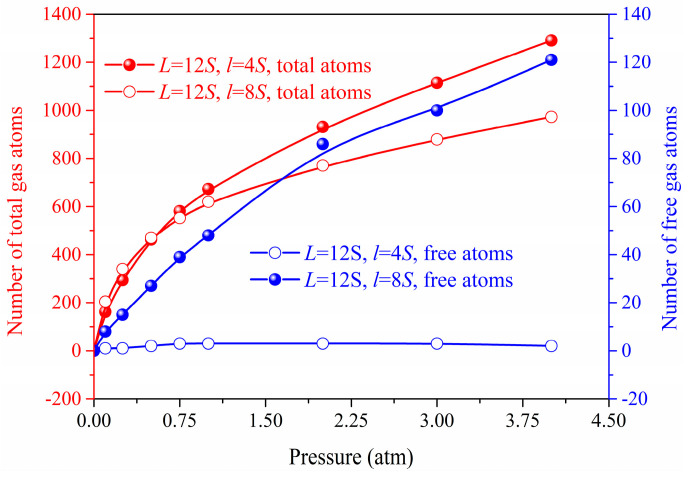
Total and free gas atoms number in the nanoporous systems.

**Figure 5:**
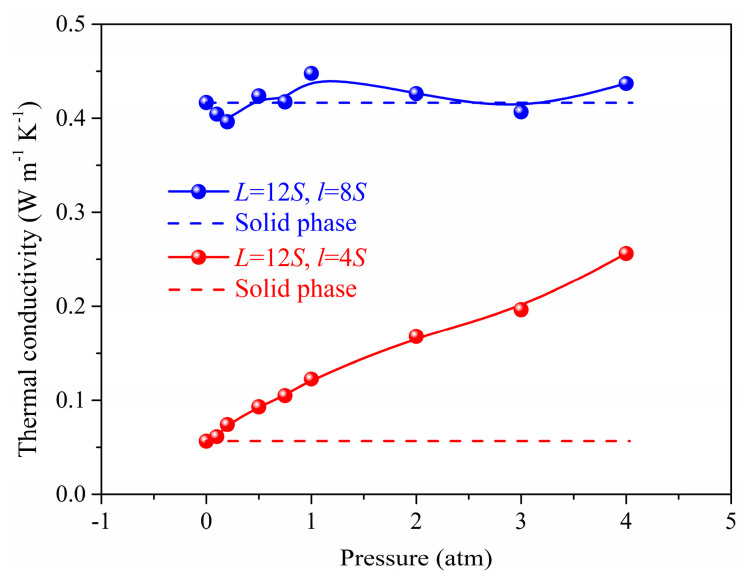
The thermal conductivity as a function of pressure.

Figure 3 and Figure 4 shows the gas atoms distribution in the nanoporous system (*L* = 12*S*, *l* = 4*S* and *L* = 12*S*, *l* = 8*S*). We use the lattice constant *S* as the cut-off radius to carry out the cluster analysis for the gas-solid system. The gas atoms near the solid phase are treated as the adsorbed state and others are considered as the free atoms. The total gas atoms in the nanoporous systems are increased with the pressure. However, for the system of *L* = 12*S*, *l* = 8*S*, the free gas atoms are not dependent on the pressure due to the space confinement. The thermal conductivity of the nanoporous system (*L* = 12*S*, *l* = 4*S* and *L* = 12*S*, *l* = 8*S*) as a function of pressure is shown in Figure 5. The total thermal conductivity of *l* = 8*S* is higher than the system of *l* = 4*S* ranging from 0 to 4 atm due to the high thermal conductivity of the solid phase (pressure = 0). For the nanoporous system of *l* = 4*S*, the total thermal conductivity increases with higher pressure or gas loadings, which is due to the increased free gas atoms corresponding to the better heat transfer capability. The obtained thermal conductivity for *l* = 8*S* is almost unchanged with the pressure. This is because there are almost no free gas atoms in the nanoporous system and the total thermal conductivity is dominated by the solid phase.

### 3.2. The Structure Effect

**Figure 6:**
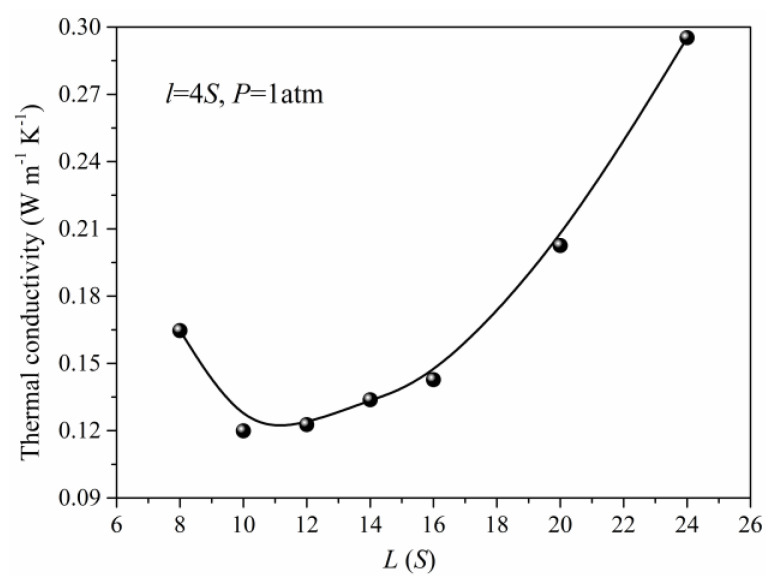
The thermal conductivity for different unit length (*L*).

**Figure 7:**
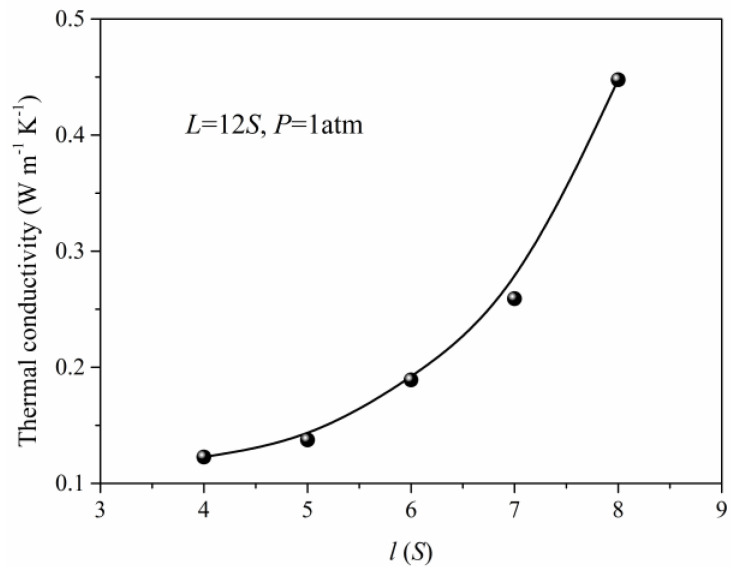
The thermal conductivity for different width (*l*).

In practical situations, the nanoporous materials have different structural features due to the preparation method and conditions. Meanwhile, the structure also has a significant effect on the total thermal conductivity of the nanoporous system. The thermal conductivity of the simulation unit adopting different structural parameters under the same pressure is obtained as shown in Figure 6 and Figure 7. When the length (*L*) of the simulation unit is increased, the thermal conductivity of the system will decrease at first and reach minimum at about 10*S*, then increase. The initial reduction of the thermal conductivity is due to the increase of porosity in which the solid phase is the main factor to determine the total thermal conductivity. As the length (*L*) of the simulation unit continues to increase, the gas atoms and the corresponding gas contributed thermal conductivity will increase. For the same length (*L*) of the simulation unit, the width increase of the solid framework will lead to the rapid increase of total thermal conductivity which is mainly attributed to the increase of thermal conductivity for solid phase. Although the gas atoms are decreased in this situation, the effect of the solid phase is more obvious and leads to the increased total thermal conductivity as shown in Figure 7.

### 3.3. Gas−Solid Interaction

**Figure 8:**
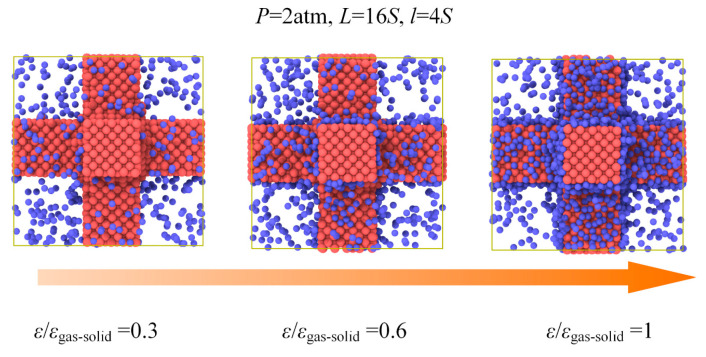
Gas atoms’ distribution for different *ε* under the pressure of 2 atm.

**Figure 9:**
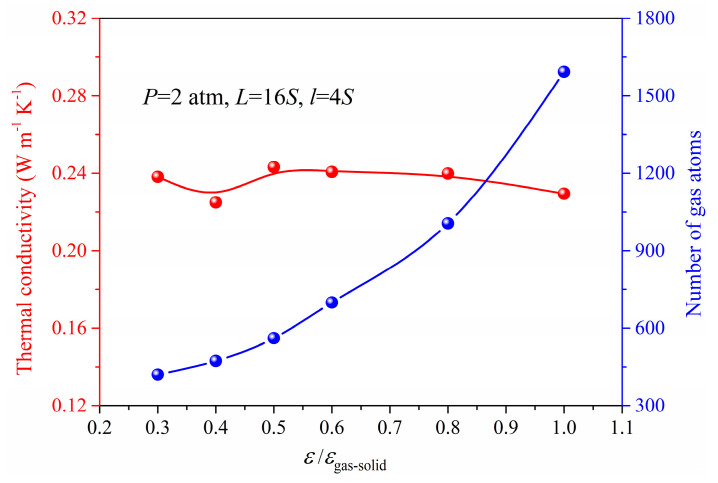
Gas atoms number and the thermal conductivity for different *ε* under the pressure of 2 atm.

In order to meet the conditions for application, the nanoporous materials may require surface modification in which situation the gas−solid interaction is changed. It is still not clear how the thermal conductivity of the nanoporous system depends on gas−solid interaction. In our simulation, the energy parameter *ε* used in Lennard−Jones 12−6 potential could be adjusted to represent this kind of surface modification as a simplification. The *ε*/*ε*_gas-solid_ ranging from 0.3 to 1 is used in the simulation under the same pressure and gas loading. Figure 8 and Figure 9 show the results of gas atoms’ distribution and the total thermal conductivity for different *ε* under the same pressure. The gas atoms in the nanoporous system are increased due to the strong gas−solid interaction and exhibit a good adsorption performance with the increased *ε*. However, as shown in Figure 9, the thermal conductivity of nanoporous system stabilizes at around 0.24 W m^−1^ K^−1^. The interaction between the solid and gas atoms shows little effect on the thermal conductivity under the same pressure. This is because although the total number of gas atoms is increased due to strong gas−solid interaction, the increased gas atoms are in an adsorbed state which has little contribution to the heat transfer in the nanoporous system. However, according to the ideal gas state equation, the free gas atoms participating in the heat conduction process are nearly identical under the same pressure. Therefore, the thermal conductivity of the nanoporous system is stable and independent of the gas−solid interaction under the same pressure.

## 4. Discussion

In a previous study [20], the heat transfer in porous crystals containing adsorbed gases was investigated. The results revealed that the thermal conductivity of the system is dominated by the thermal conductivity of the crystal and reduced as the concentration of gas in the pores increases. The decreased conductivity with increased gas concentration is due to the phonon scattering of the crystal caused by the interaction with gas atoms. In our simulation, similar results are not found for the porous crystal system. Therefore, in such systems, the total thermal conductivity is the result of multiple factors including gas concentration, diffusivity, material properties, structural parameters, and so on. The effect of pressure on the total thermal conductivity presents different characteristics for the nanoporous system.

**Figure 10:**
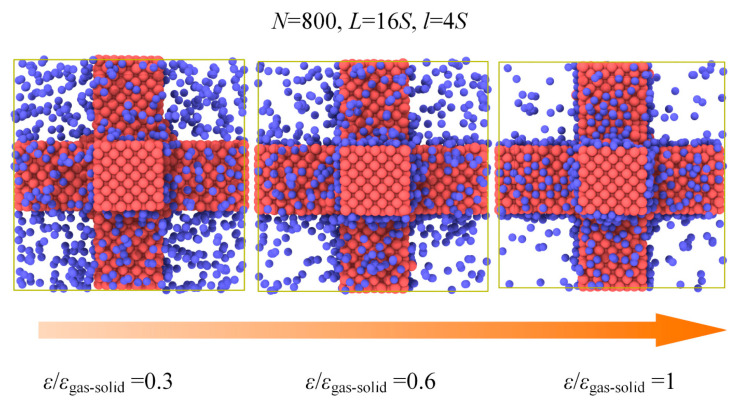
Gas atoms distribution for different *ε* under constant gas atoms number.

**Figure 11:**
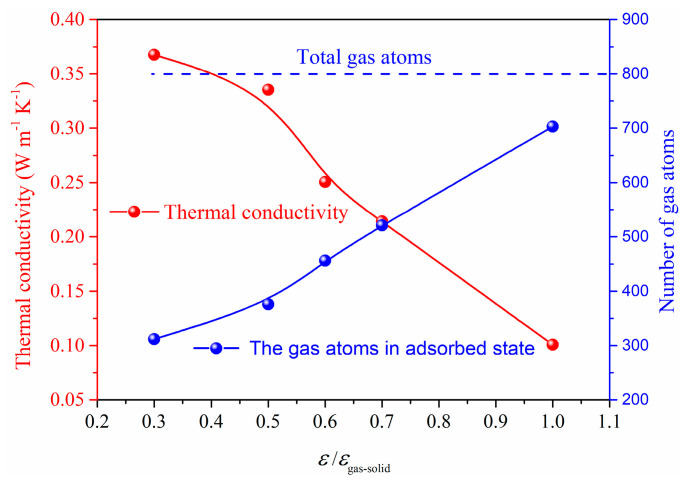
Absorbed atoms and thermal conductivity for constant gas atoms.

To further illustrate the effect of gas−solid interaction on the total thermal conductivity, the case of constant gas atoms for different gas−solid interactions is investigated as shown in Figure 10 and Figure 11. In the system of *L* = 16*S*, *l* = 4*S*, we can find that the gas atoms for *ε*/*ε*_gas-solid_ = 0.3, which means a lower surface energy, could maintain a free state. However, for the case of *ε*/*ε*_gas-solid_ = 1, most gas atoms are limited on the surface and keep the absorbed state which cannot contribute to the heat transfer in the porous structures coupling gas and solid phases. Therefore, the total thermal conductivity exhibits a dramatic decrease from 0.37 W m^−1^ K^−1^ to 0.11 W m^−1^ K^−1^ due to the different states of the gas atoms. The adsorption effect on the total thermal conductivity under constant gas atoms is remarkable, and the free gas atoms are essential for the heat transfer in the porous structures coupling gas and solid phases.

## 5. Conclusions

In this work, we investigated the thermal conductivity of the ordered porous structures using the Grand Canonical Monte Carlo method (GCMC) and molecular dynamics simulation (MD). The effect of gas concentration, porous structure, and gas−solid interaction on thermal conductivity is revealed.

The simulation results show that for different structures, the pressure effect on thermal conductivity exhibits an inconsistent mode. Under the same pressure, the thermal conductivity is barely changed for different interactions between gas and solid phases. Furthermore, the state of gas atoms, absorbed or free, has a significant impact on the thermal conductivity of the nanoporous system. From this study, the heat transfer in porous structures coupling gas and solid phases could be understood on a fundamental level.

## Figures and Tables

**Table 1:** The parameters used in the simulation.

Material	*ε* (kcal/mol)	*σ* (Å)	*l* (*S*)	*L* (*S*)
Argon	0.39	3.35	4, 5, 6, 7, 8	8, 12, 16, 20, 24
Helium	0.25	6.70		
Argon-Helium	0.16	10.05		

## Data Availability

The data presented in this study are available on request from corresponding author.
